# Resolution of Osseous Sarcoidosis with Methotrexate

**DOI:** 10.1155/2019/4156313

**Published:** 2019-11-11

**Authors:** Christopher Kanner, Bonita Libman, Morgan Merchand, Diego Lemos

**Affiliations:** University of Vermont Medical Center, The Robert Larner, M. D. College of Medicine at the University of Vermont, Burlington, VT, USA

## Abstract

Though a relatively uncommon manifestation of sarcoidosis, some clinicians are tasked with managing osseous involvement of disease, and the optimal treatment approach in this setting is not well established. Previous studies have shown variable efficacy for osseous sarcoidosis utilizing multiple agents alone or in combination, often using imaging follow-up in conjunction with clinical assessment to evaluate response to treatment. We present a case of widespread skeletal involvement of sarcoidosis without evidence of concurrent pulmonary disease demonstrating marked clinical improvement and near-complete resolution of imaging abnormalities on magnetic resonance imaging (MRI) following the use of methotrexate as the primary pharmacologic agent.

## 1. Introduction

Sarcoidosis is a multisystem inflammatory granulomatous disease that can affect any organ [1]. Tissue biopsy typically demonstrates noncaseating granulomas. Lungs, lymph nodes, eyes, liver, spleen, and skin are the most common sites of involvement [2]. Osseous sarcoidosis has been less commonly reported in 1–13% of affected patients [2, 3, 4], with some higher estimates that include asymptomatic, incidentally detected osseous lesions. The small bones of the hands and feet have been described as the most common sites of bony involvement; however, a large series of sarcoidosis patients reviewed for the presence of skeletal disease [5] suggests that the pelvis and lumbar spine may be the most common sites of osseous infiltration.

The spectrum of imaging appearances of osseous sarcoidosis has been well documented. The presence of classic lesions in the small bones of the hands and feet can typically be ascertained utilizing conventional radiographs in the proper clinical context with lesions demonstrating a characteristic “lacy” lytic appearance [6], whereas lesions in larger bones may be lytic, sclerotic, permeative, or undetectable at radiography. Variable MRI appearances have been described, including round intramedullary lesions, areas of confluent marrow replacement, patchy diffuse intramedullary lesions, and a pattern of more ill-defined discrete lesions described as having a “starry sky” appearance. Generally, sarcoid lesions show low signal intensity on T1-weighted imaging, high signal intensity on T2-weighted or proton-density-weighted fat-saturated images, and varying degrees of enhancement following contrast administration [6]. Neither the MRI nor the radiographic appearances are distinguishable from a number of other marrow replacing processes which include metastatic disease, lymphoma, multiple myeloma, and some infectious etiologies. Sarcoid lesions of bone may resolve on follow-up imaging, sometimes marked by the presence of residual “ghosts” produced by the presence of intramedullary fibrosis and/or fat at the sites of previous disease.

Treatment of skeletal sarcoidosis is not standardized due to a lack of guidelines from the literature and due to variability in clinical course including reported cases of improvement with no treatment at all [7]. Nonsteroidal anti-inflammatory drugs (NSAIDs), corticosteroids, methotrexate (MTX), hydroxychloroquine (HCQ), and tumor necrosis factor-alpha (TNF-alpha) inhibitors are possible pharmacologic options [5, 8] among others. A recent retrospective French multicenter review analyzing treatment response in patients with biopsy-proven sarcoidosis and osseous manifestations reported response rates with glucocorticoids alone at 52% (23/44), glucocorticoids plus MTX at 69% (9/13), and glucocorticoids plus HCQ at 67% (4/6), as well as response rate for TNF-alpha inhibitors at 100% (10/10) [9]. Most additional support for specific treatment regimens comes in the form of case series or case reports, the largest of which included 9 cases of symptomatic skeletal disease treated with varying combinations of prednisone, MTX, HCQ, or TNF-alpha inhibitors in refractory cases, with 67% (6/9) of these patients endorsing resolution of symptoms at time of last follow-up. Several additional single case reports have demonstrated clinical and/or radiographic improvement of osseous sarcoid lesions utilizing a variety of treatment approaches most commonly with corticosteroid therapy and/or a disease-modifying antirheumatic drug such as MTX [10–14].

## 2. Case Presentation

A 45-year-old male presented to the emergency department with several weeks of slowly progressive dull right lower abdominal and groin pain. The right groin pain was worse with activity and weight-bearing, and partially responsive to ibuprofen. He also had a 15- to 20-year history of chronic right-sided low back pain due to degenerative disk disease at L5-S1, and reported a mild increase in this pain concomitant with the groin pain. He had no other medical problems. His musculoskeletal physical examination revealed right paralumbar pain with forward flexion but no spine tenderness, full and painless range of motion of both hips, and bilateral groin tenderness to palpation without any lymphadenopathy. His general examination was notable only for a palpable liver edge but no palpable splenomegaly. Laboratory evaluation at presentation revealed normal or negative electrolytes, calcium, creatinine, transaminases, hemoglobin, platelet count, rheumatoid factor, sedimentation rate, C-reactive protein, and urinalysis. The total alkaline phosphatase was elevated at 170 U/L (upper limit normal 126), 1,25-dihydroxycholecalciferol was elevated at 65 pg/mL (upper limit normal 64), angiotensin-converting enzyme was elevated at 101 U/L (upper limit normal 53), and leucocyte count was low at 3.39 K/cm^2^ (lower limit normal 4).

**Figure 1:**
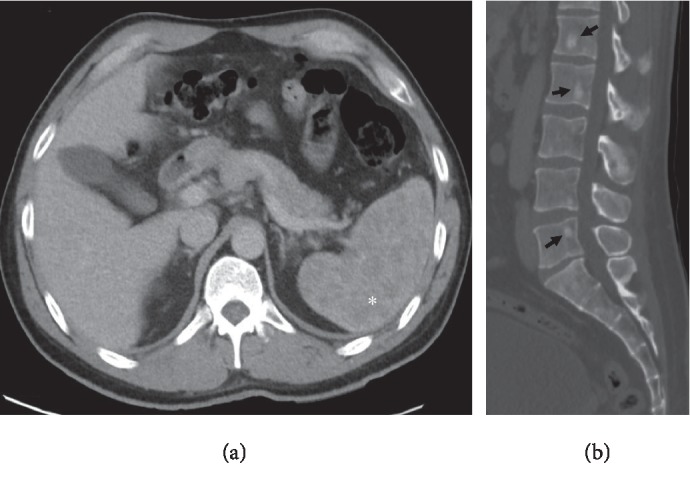
Contrast-enhanced CT of the abdomen pelvis demonstrates an enlarged and heterogeneous spleen (asterisk (a)) and multiple nonspecific sclerotic osseous lesions within the lumbar spine and sacrum (arrows (b)).

Contrast-enhanced computed tomography (CT) images of his abdomen and pelvis revealed an enlarged and heterogeneous spleen as well as numerous small nonspecific primarily sclerotic osseous lesions in his lower thoracic spine, lumbar spine, and pelvis without additional CT findings of concern (Figure 1). Differential considerations based on the CT findings included lymphoma, metastatic disease, and sarcoidosis. CT-guided percutaneous needle core biopsy and cytology of splenic tissue revealed nonnecrotizing and noncaseating granulomatous inflammation with multinucleated giant cells, consistent with sarcoidosis. Stains and cultures for acid-fast bacilli, fungi, and bacteria were negative. Flow cytometry for clonal cell population was negative.

**Figure 2:**
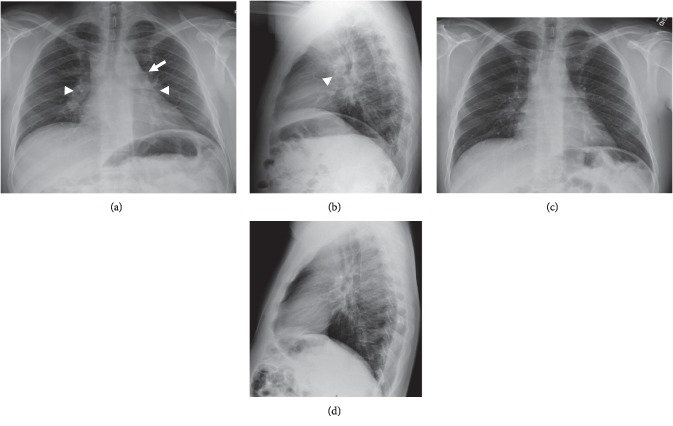
PA and lateral chest radiographs obtained 5 years earlier (a, b) show mild convexity at the AP window (arrow) and subtle perihilar fullness (arrowheads) which in retrospect may have corresponded to lymphadenopathy rather than enlarged pulmonary arteries as initially interpreted. However, chest radiographs at current presentation (c, d) show no evidence of pulmonary or mediastinal disease.

A chest radiograph performed in the emergent setting approximately five years earlier for an episode of chest pain was interpreted at that time as being notable only for an abnormal convexity in the region of the aortopulmonary window, likely due to a mildly enlarged main pulmonary artery (Figure 2). A retrospective review of that chest radiograph following the new diagnosis of sarcoidosis suggested that the previously described abnormal contour in the AP window, along with mild bilateral perihilar fullness, may have corresponded to lymphadenopathy rather than an enlarged pulmonary artery; however, no cross-sectional imaging was obtained for confirmation at that time, and therefore the possibility of prior lymphadenopathy in the chest remains uncertain. However, a new chest radiograph performed at the time of current presentation revealed neither residual mediastinal contour abnormalities nor perihilar fullness to suggest active pulmonary or mediastinal involvement of disease (Figure 2). Additionally, a cardiac MRI performed concurrently was unremarkable (not pictured).

**Figure 3:**
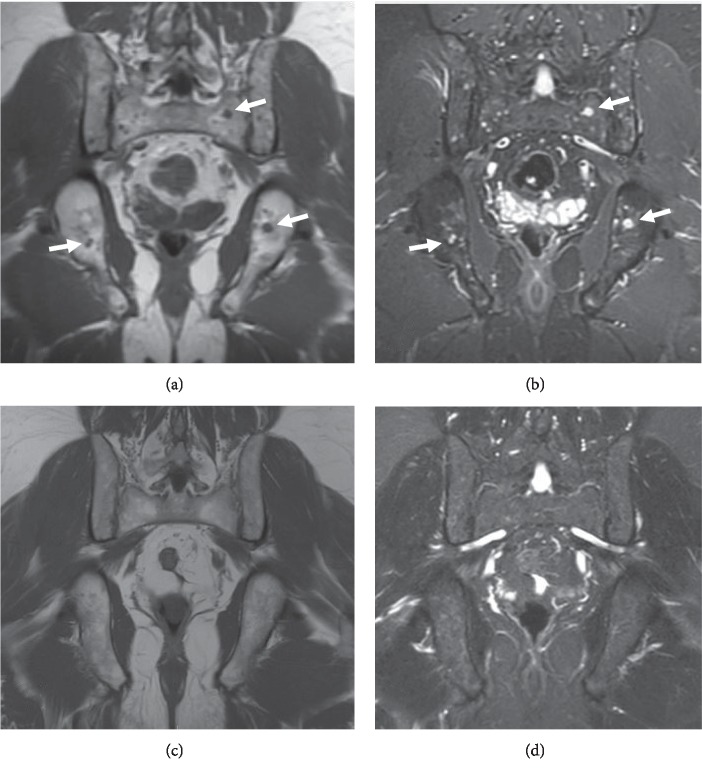
T1-weighted (a, c) and STIR (b, d) coronal MR images of the pelvis prior to treatment (top row) and following treatment (bottom row) show numerous small intramedullary foci of low signal on T1 and high signal on STIR (representative arrows), which essentially resolve following treatment.

MRI of the pelvis and contrast-enhanced MRI of the lumbar spine and right hip were obtained to evaluate the osseous lesions identified on CT and to further elucidate the source of pain localizing to the right hip, groin, and low back. MR images showed innumerable small enhancing intraosseous lesions throughout the pelvis (Figure 3), lower thoracic spine, and lumbar spine, findings favored to correspond to polyostotic skeletal involvement of sarcoidosis given the clinical scenario.

The patient was treated with 40 mg of prednisone orally for 3 days with a taper by 10 mg every 3 days until off, with improvement in symptoms, but recurrence of right groin pain off prednisone. He was then started on MTX 15 mg by mouth weekly. On follow-up five months later, his abdominal pain had resolved, and groin pain had improved, but not resolved. He received an injection of triamcinolone acetonide to the right hip joint without improvement. The dose of MTX was increased to 20 mg weekly, and three months later, the patient reported further improvement in groin and hip pain, allowing him to be physically active without any limitations.

**Figure 4:**
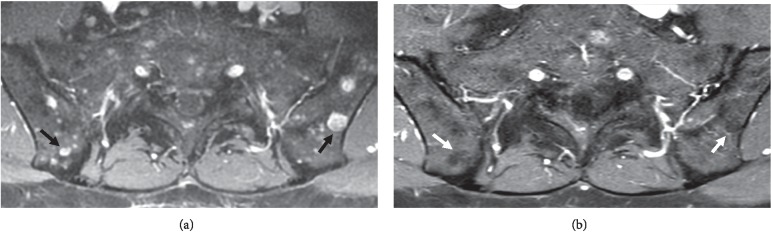
Contrast-enhanced T1 fat-saturated axial images at the level of the SI joints prior to (a) and following (b) treatment show characteristic low signal “ghosts” (white arrows (b)) at sites of prior-enhancing osseous lesions (black arrows (a)) indicating fibro-fatty tissue at these locations.

Approximately 10 months following the initial MRI and 9 months following the initiation of MTX, a repeat contrast-enhanced MRI of the pelvis and lumbar spine demonstrated a marked reduction in quantity, size, and degree of enhancement associated with the previously described innumerable intraosseous lesions in the pelvis and spine, imaging features consistent with significant regression of skeletal sarcoidosis (Figure 3). The presence of aforementioned low signal “ghosts” could be observed at sites of previously evident disease foci (Figure 4). Furthermore, the marked imaging regression of skeletal disease correlated with a substantial clinical reduction in right hip, groin, and lower back pain symptoms which were considered likely to be attributable to the presence of extensive osseous sarcoid lesions in these regions.

## 3. Discussion

We report a case of near-complete resolution of widespread osseous sarcoidosis with documentation of disease regression on MRI in a patient receiving methotrexate monotherapy following a short prednisone taper. This patient presented initially with groin pain and was shown to have osseous disease based on a CT obtained in the emergent setting which was later confirmed with contrast-enhanced MRI. The diagnosis of sarcoidosis was established by biopsy of the patient's enlarged spleen that showed nonnecrotizing granuloma formation. There was no concurrent evidence of extraskeletal disease aside from splenomegaly; specifically, chest radiograph and cardiac MRI showed no evidence for sarcoidosis. Furthermore, the patient was otherwise asymptomatic aside from hip and groin pain. The patient was started on MTX for osseous sarcoidosis, and approximately 9 months following initiation of therapy, there was a substantial improvement of his pain. Follow-up MR imaging demonstrated evidence of significant reduction in skeletal disease burden.

As mentioned previously, there are no standard guidelines available regarding treatment of osseous sarcoidosis. Based on published reports and the authors' experiences, a general approach to treatment can include corticosteroids, which have shown good efficacy and are the most common first line treatment option. DMARDs such as HCQ or MTX can be used alone for maintenance therapy or in conjunction with steroids in those patients who are refractory or unable to successfully wean off steroids. The choice between these two DMARDs depends upon severity of disease and patient factors that might influence the risk of side effects. Specifically, HCQ can be effective in milder disease though often takes a longer time to achieve treatment response and carries rare ocular toxicity and therefore may be contraindicated in patients with pre-existing ocular pathology. MTX is considered a stronger and faster-acting agent though it carries a wider side effect profile which includes possible liver toxicity and myelosuppression. Other therapies including alternative DMARDs or immunosuppressives such as leflunomide, azathioprine, or mycophenolate mofetil may be required if HCQ or MTX is not effective or poorly tolerated. Finally, TNF-alpha inhibitors can be used when DMARD therapy fails.

Our case is unique and instructive for two main reasons. The first is the rare nature of osseous and splenic involvement of sarcoidosis in the absence of any concurrent pulmonary disease. The second is the evidence of essentially complete resolution of widespread osseous sarcoidosis treated with MTX and documented with follow-up MRI, corresponding with marked clinical improvement of symptoms. This report serves as an affirmation of the efficacy of methotrexate for treatment of osseous sarcoidosis and emphasizes the utility of follow-up MR imaging to document treatment response.
